# Case Report: A severe case of human parechovirus encephalitis in a neonate

**DOI:** 10.3389/fped.2026.1889734

**Published:** 2026-10-09

**Authors:** A. M. Grimm, A. Heep, Christiaan Peter Sentner, K. M. Fiedler

**Affiliations:** 1 University Hospital of Paediatrics – Elisabeth Children’s Hospital, Department of Neonatology, Paediatric Intensive Care, Paediatric Cardiology, Paediatric Pneumology and Allergology, Klinikum Oldenburg AöR, Oldenburg, Germany; 2 Carl von Ossietzky Universität Oldenburg, Faculty VI- Medicine and Health Sciences, Oldenburg, Germany

**Keywords:** case report, encephalitis, neonatal, parechovirus, seizures

## Abstract

**Introduction:**

Human parechovirus is a common pathogen causing respiratory and gastroenterological symptoms. Symptoms range from asymptomatic courses to rare encephalitis or sepsis-like symptoms. Studies regarding neurodevelopment after parechovirus encephalitis in infants are conflicting with indications of possible short or long term neurodevelopmental impairments. In this case report we describe an especially severe course of human parechovirus infection.

**Case:**

A two-week-old infant presented with fever and developed seizures and apneic episodes after admission. Laboratory tests revealed leukopenia. A lumbar puncture revealed parechovirus via polymerase chain reaction (PCR). Recurrent seizures required extensive anticonvulsive therapy. Invasive ventilation for four days was necessary due to apneic episodes. Cranial magnetic resonance imaging (MRI) revealed white matter involvement.

**Discussion:**

In neonates with a sepsis-like presentation and largely unremarkable laboratory and cerebrospinal fluid findings, parechovirus encephalitis is an important differential diagnosis. In this case, follow-up examinations showed age-appropriate neurodevelopment. However, owing to the severe clinical presentation and extensive neuroimaging abnormalities, long-term neurodevelopmental monitoring is warranted.

## Introduction

Human parechovirus (HPeV) is a common pathogen belonging to the family of picornaviridae, which also includes other virus types such as enterovirus or poliovirus and is associated especially with respiratory and gastrointestinal symptoms. In most cases, infection is mild, and the majority of children have been infected by the age of three years (1). Among young infants presenting with fever without an identifiable source upon initial clinical assessment, HPeV is later detected in 6%–15% of cases (2, 3). Several studies have reported a male predominance, with male infants accounting for 56%–91% of cases (4).

Among the 19 recognised HPeV genotypes, types 1 and 3 in particular have been associated with more severe disease (5). HPeV type 3 is the genotype predominantly involved in severe infections, especially among infants younger than three months. Whereas the clinical spectrum of parechovirus infection ranges from asymptomatic infection to rare cases of encephalitis, severe manifestations occur predominantly in this age group (4–7). Fever is the most common clinical manifestation, occurring in 80%–100% of reported cases. Erythema is reported in 20%–60% of cases, whereas seizures occur in 3%–65%. Other manifestations include diarrhea, respiratory symptoms, irritability, poor feeding and tachycardia (4).

Laboratory abnormalities are generally mild and non-specific, with leukopenia being particularly common in infants younger than three months. Cerebrospinal fluid (CSF) findings are usually unremarkable, and pleocytosis is typically absent. Cranial ultrasonography is generally normal. In patients with neurological involvement, MRI is the imaging modality of choice and may reveal characteristic white-matter abnormalities (4).

In a British study by Braccio et al., 83% of patients with HPeV infection admitted to a paediatric intensive care unit (PICU) were younger than one year, and 40% presented with neurological manifestations. Neurological complications were particularly common among infants younger than three months, occurring in 46% of this age group. Invasive mechanical ventilation was required in 77% of patients admitted to the PICU. The mortality rate was approximately 8% (8). In other studies among infants with severe neurological manifestations of HPeV type 3 infection, reported mortality rates range from 1.6% to 6% (5).

Here, we report an exceptionally severe case of HPeV infection in a young infant.

## Case description

### Medical history

A two-week-old male infant presented to our emergency department with fever. His stools had been slightly looser than usual; however, oral intake remained adequate. Several family members were experiencing symptoms of an upper respiratory tract infection at the time of presentation. He had been born by uncomplicated spontaneous vaginal delivery at 37 + 2 weeks of gestation, with a birth weight of 3,300 g. Maternal group B streptococcus colonisation status was unknown. The infant had otherwise been healthy and was receiving routine vitamin D supplementation.

### Clinical findings

On admission, the infant was in an age-appropriate nutritional state but appeared generally unwell. His skin was pale pink, with mottling of the limbs. The anterior fontanelle was soft and flat. Heart sounds were normal and regular. Respiratory, abdominal, and ear, nose, and throat examinations were unremarkable.

On neurological examination, the infant was somnolent but arousable and exhibited a high-pitched cry. Spontaneous movements, muscle tone, and strength were symmetrical.

Because of the fever, the patient was initially admitted to the general paediatric ward for clinical observation. During the first hours of his inpatient stay, he developed recurrent apnoeas and seizures and was therefore transferred to the PICU.

Following transfer to the PICU, the recurrent seizures were characterised more precisely as predominantly unilateral twitching of the upper limbs, occasionally accompanied by lip smacking. Individual episodes lasted 10–30 s and were associated with apnea and oxygen desaturation.

During hospital stay, the infant developed a generalised erythematous rash.

## Timeline during the inpatient stay

See Figure 1.

**Figure 1:**
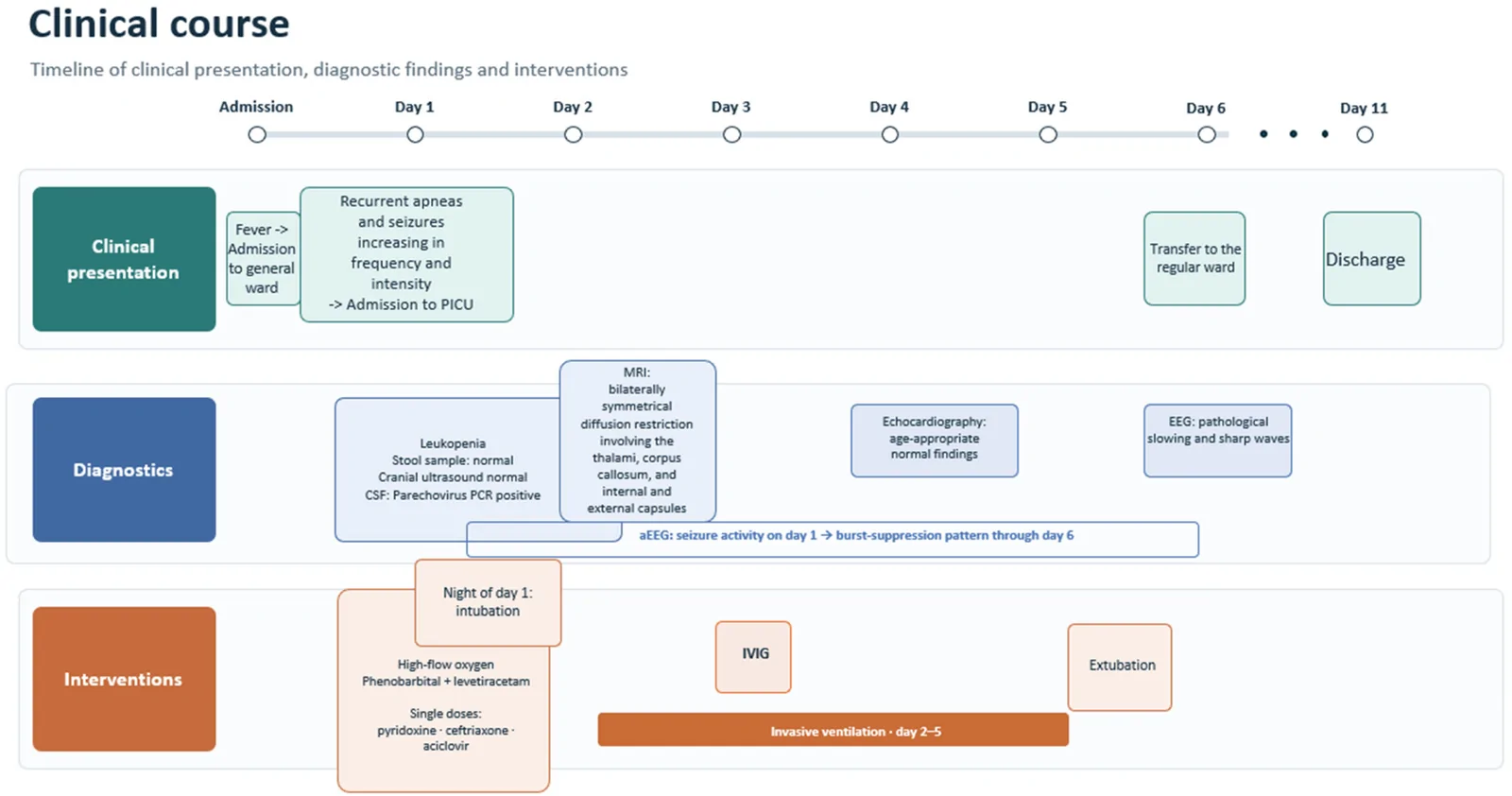
Timeline of the clinical course during the inpatient stay [aEEG-(amplitude-integrated) electroencephalography; MRI: magnetic resonance imaging; CSF- cerebrospinal fluid; EEG- electroencephalogram; IVIG- intravenous immunoglobulin; PICU- paediatric intensive care unit]*.*

### Diagnostic assessment, therapy and follow-up

#### Laboratory findings

Laboratory investigations revealed leukopenia with lymphopenia and a mildly elevated interleukin-6 (IL-6) concentration. Mild hypernatremia and a slight elevation in serum bilirubin were also noted. The remaining serum electrolytes, liver and renal function parameters were within the reference ranges (Table 1).

**Table 1:** Pathological laboratory findings*.*

Parameter	Norm	Result
Leukocytes	7,8–15,91 10E9/L	4,19 10E9/L
Neutrophiles	14–55%	45%
Lymphocytes	34–77%	30%
Monocytes	4–18%	23%
Eosinophiles	<5%	1%
Basophiles	<1%	0%
IL-6	<7 ng/L	84 ng/L
Natrium	136–145 mmol/L	152 mmol/L
Bilirubin	<1,2 mg/dL	4,5 mg/dL

Stool PCR testing was negative for adenovirus, astrovirus, norovirus, sapovirus, and rotavirus.

Metabolic test for vitamin B6 dependent epilepsy was negative.

#### CSF findings

Because of the recurrent seizures, a lumbar puncture was performed. Routine CSF analysis was unremarkable, with a leukocyte count of 1 cell/µL (reference range, 0–5 cells/µL). Multiplex PCR analysis of the CSF was positive for human parechovirus.

#### Imaging

Initial cranial ultrasonography was unremarkable. Brain MRI subsequently demonstrated extensive, bilateral symmetrical diffusion restriction involving the thalami, corpus callosum, and internal and external capsules (Figure 2). This pattern was consistent with severe human parechovirus encephalitis.

**Figure 2:**
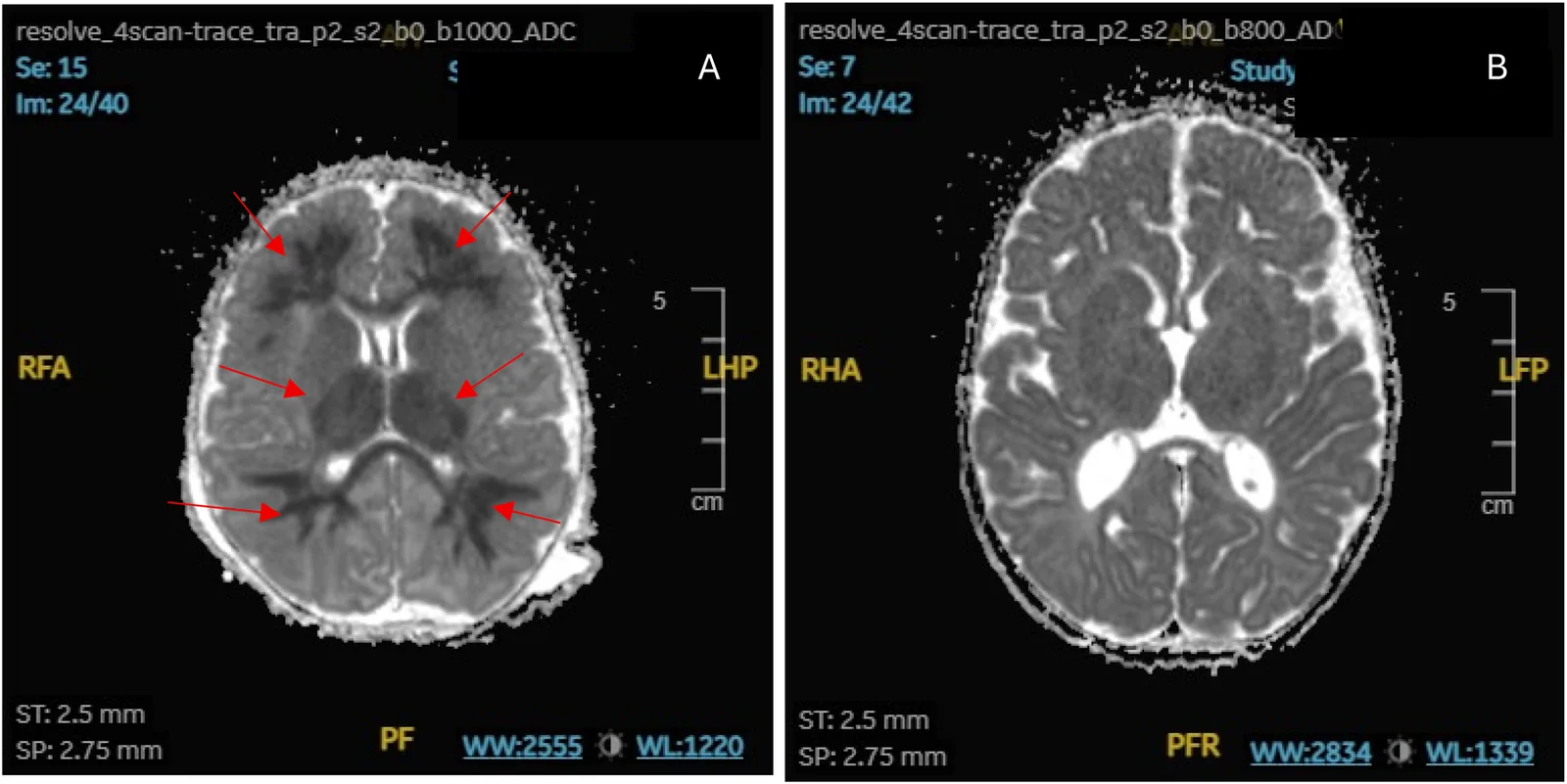
Brain magnetic resonance imaging during the acute phase and at three-month follow-up. **(A)** Initial diffusion-weighted magnetic resonance imaging (02/2025) demonstrating extensive bilateral and symmetrical diffusion abnormalities involving the thalami, corpus callosum, and internal and external capsules (red arrows). **(B)** Follow-up magnetic resonance imaging three months after discharge showing reduced supratentorial volume, predominantly affecting the white matter, and poor delineation of the corpus callosum. The previously observed diffusion abnormalities had resolved.

Echocardiography showed normal cardiac morphology, dimensions and function.

#### EEG

On the day of admission, amplitude-integrated electroencephalography (aEEG) demonstrated recurrent seizures with varying localisation, some of which were accompanied by clinical manifestations. The background activity was characterised by a persistent burst-suppression pattern.

#### Treatment and course

After the onset of seizures, anticonvulsive treatment was initially commenced with pyridoxine (a single dose of 25 mg/kg) and phenobarbital. Because of persistent seizures, levetiracetam was subsequently added; however, adequate seizure control was only achieved with substantial delay. After the positive HPeV PCR result became available, pyridoxine was discontinued.

The intravenous phenobarbital dose was increased to 8 mg/kg once daily. Levetiracetam was initiated at a dosage of 20 mg/kg intravenously twice daily.

Empirical antimicrobial treatment for suspected meningitis/encephalitis was initiated with ceftriaxone (100 mg/kg once) and aciclovir (20 mg/kg once). Both agents were discontinued after the CSF results became available.

Despite high-flow nasal oxygen therapy, recurrent apneic episodes requiring bag-mask ventilation occurred. The patient was therefore intubated on the first day of admission to the PICU. Invasive mechanical ventilation was continued for four days, and the patient was extubated following improvement of the aEEG findings. No further clinical seizures occurred during the hospital stay.

During mechanical ventilation, the patient received a single intravenous dose of immunoglobulin (IVIG) at 1.5 g/kg because of the severity of the clinical presentation.

After extubation, phenobarbital was continued at 4 mg/kg orally once daily and levetiracetam was continued orally at 30 mg/ kg twice daily.

After discharge from the PICU, the patient subsequently remained on the general paediatric ward for six days. EEG demonstrated independent multifocal epileptiform sharp waves at both frontal regions, with right-sided predominance and activation during sleep.

The patient was discharged on maintenance anticonvulsive therapy with phenobarbital and levetiracetam and received outpatient physiotherapy. At follow-up one month after discharge, EEG continued to demonstrate sharp waves.

A further assessment three months after discharge, including brain MRI, revealed reduced supratentorial volume, predominantly affecting the white matter, and poor delineation of the corpus callosum (Figure 2). The previously observed white-matter diffusion restriction had resolved. Clinically, there was no evidence of relevant developmental delay.

At the three-month follow-up, the patient had remained seizure-free for 11 weeks and the EEG was age-appropriate. Anticonvulsive medication was therefore discontinued. Another assessment at six months after discharge showed age-appropriate development of the patient and a normal EEG. Further follow-up assessment is scheduled for the age of 18 months to monitor his neurocognitive and developmental outcome.

## Discussion

This case illustrates an exceptionally severe course of neonatal human parechovirus infection. However, the findings are inherently limited by the single-patient nature of a case report. Moreover, the follow-up period of only six months precludes conclusions regarding the patient's long-term neurological and developmental outcome.

HPeV is transmitted via the faecal–oral and respiratory routes, particularly through close contact within households (7). In the present case, several close family members had symptoms of an upper respiratory tract infection, suggesting possible household transmission. However, as the family members were not tested for HPeV, the source and route of transmission could not be definitively established.

The patient exhibited manifestations resembling those reported in other severe cases of HPeV infection. HPeV infections can cause a broad spectrum of symptoms depending on the viral genotype, ranging from fever and gastrointestinal or respiratory symptoms to sepsis-like illness, (meningo-) encephalitis, seizures, apnea, rash, myocarditis, hepatitis, and lethargy. Severe manifestations have been attributed, at least in part, to a systemic hyperinflammatory immune response. Viraemia may occur more frequently in young infants because of low levels of maternally derived antibodies and immaturity of the neonatal immune system. In addition, the greater permeability of the blood–brain barrier in young infants may facilitate viral neuroinvasion (7, 9, 10).

Enteroviruses belong to the same viral family as HPeV and may cause a similar spectrum of disease, including fever, respiratory and gastrointestinal symptoms, sepsis-like illness, (meningo-) encephalitis, hand, foot and mouth disease, herpangina, rash, myocarditis and hepatitis (11).

Especially the generalized erythematous rash which our patient exhibited is a typical sign of HPeV infection, particularly reported in type 3 (10). In Shoji et al, 80% of neonates and infants with HPeV type 3 infection showed palmar and plantar rash, 58% with inclusion of trunk and limbs (12).

HPeV type 3 is known to cause especially severe symptoms in young infants. A study of Hara et al. suggests these sepsis-like symptoms may be caused by an haemophagocytic lymphohistiocytosis (HLH)-like disease due to an aberrant immune response. HLH is a severe hyperinflammatory syndrome due to immune dysregulation marked by elevated ferritin and cytopenia. Hyperferritinaemia and cytopenia are described to indicate hypercytokinemia. Hara et al. demonstrated elevated ferritin levels in infants with HPeV type 3 infection on day four to five in comparison to patients with enterovirus infection. Ferritin concentrations were higher in infants with apnea than in those without (13). Although the precise mechanisms underlying the particularly severe manifestations of HPeV type 3 infection in young infants remain unclear, these findings suggest that an HLH-like immune response may contribute to disease severity in this age group.

On the other hand, another case series regarding neonates and infants with sepsis and HLH-like illness in HPeV type 3 infection showed elevated aspartate aminotransferase (AST), soluble interleukin-2 receptor and lactate dehydrogenase (LDH) in all cases (14).

AST and LDH were normal in our patient while we did not measure ferritin and the soluble interleukin 2 receptor. This suggests that in cases of severe presentation of HPeV infection, laboratory measurements should include indicators of HLH-like illness. The specific genotype would have been of interest in this case as well, in order to help clarify the cause of this particularly severe course. As described above, the patient's rash and sepsis-like presentation in combination with encephalitis might indicate an infection with HPeV type 3.

Apart from leukopenia, the patient exhibited no clinically significant laboratory abnormalities. This paucity of laboratory findings is consistent with previous reports. CSF pleocytosis is absent in most cases of HPeV infection, whereas leukopenia is a commonly reported finding (6, 7, 9, 15).

At our institution, PCR testing for HPeV is not currently available for serum samples. More widespread availability of blood-based HPeV PCR testing could facilitate earlier diagnosis and potentially allow earlier discontinuation of empirical antibiotic treatment in young infants. As in our case, an important differential diagnosis is bacterial meningo-/ encephalitis which is why antibiotic treatment (ceftriaxone) was administered until receiving positive CSF PCR for HPeV and negative PCR for bacterial pathogens such as pneumococcus, meningococcus and haemophilus influenzae. In the present case, ceftriaxone was selected as the first-line empirical antibiotic until receiving these findings despite the accompanying gastrointestinal symptoms, as the clinical presentation raised concern for meningoencephalitis. This management was in accordance with our institution's treatment protocol.

At our institution, HPeV is not included in the routine multiplex viral stool PCR panel; consequently, the stool sample could not be tested for HPeV. Expanding the range of pathogens included in such panels may improve the detection rate of viral infections in young infants.

The literature supports collecting respiratory tract, stool, blood, and CSF samples for PCR testing to facilitate the early detection of viral pathogens, particularly HPeV (7). Broader access to appropriate molecular diagnostic testing may therefore improve the timely diagnosis of HPeV infection (7, 9).

The diagnosis of HPeV encephalitis was primarily established by detection of viral RNA in the CSF using PCR. However, the assay used in this case did not determine the specific HPeV genotype.

Overall, brain MRI is the imaging modality of choice for detecting white-matter abnormalities in patients with HPeV encephalitis. The extensive diffusion restriction observed on diffusion-weighted imaging (DWI) in our patient represents a characteristic neuroimaging pattern of HPeV encephalitis (7).

Herpes simplex virus (HSV) encephalitis represents an important differential diagnosis in neonates presenting with a sepsis-like clinical picture and focal seizures. Empirical treatment with aciclovir was therefore initiated in the present case. Neuroimaging can assist in distinguishing these conditions: HPeV encephalitis typically produces extensive, bilateral symmetrical white-matter diffusion restriction, whereas HSV encephalitis more commonly involves cortical and temporal regions. The predominantly white-matter pattern observed in our patient was therefore consistent with HPeV encephalitis (6, 15).

Given the potentially severe consequences of untreated bacterial meningitis and herpes simplex virus encephalitis, empirical antibacterial and antiviral treatment was initiated immediately. This approach is consistent with recommendations to commence empirical anti-infective therapy in infants with suspected meningoencephalitis and to discontinue treatment once HPeV infection has been confirmed and bacterial and HSV infection have been excluded (4).

Until the positive PCR result was received, the patient was given pyridoxine due to the differential diagnosis of vitamin B6-dependent epilepsy, which is rare but significant at this age. Neonates with this diagnosis show treatment-resistant seizures with various semiologies abnormal aEEG findings, such as a burst-suppression pattern. In vitamin B6-dependent epilepsy, early metabolic testing and treatment with pyridoxine are essential (16, 17).

Other differential diagnoses of neonatal seizures include hypoxic–ischaemic encephalopathy, intracranial haemorrhage, cerebral infarction, disturbances of glucose or electrolyte homeostasis, congenital metabolic disorders, genetic epilepsies, haemodynamically significant congenital heart disease, and intoxication (18). The clinical examination, laboratory findings, microbiological results, echocardiography and neuroimaging did not provide evidence of the alternative diagnoses.

As no specific antiviral therapy is currently available for HPeV infection, management is primarily supportive and directed towards acute complications. In the present case, recurrent apnea required intubation and mechanical ventilation.

The use of intravenous immunoglobulin (IVIG) and corticosteroids, including methylprednisolone, has been reported in individual cases of encephalitis; however, evidence supporting their efficacy remains limited (4, 6). IVIG has primarily been used in severe HPeV infections complicated by myocarditis or fulminant sepsis. Brisca et al. noted that its potential efficacy may depend on the presence and concentration of HPeV-specific neutralising antibodies in donor plasma. Antibody titres against HPeV type 3 may be low because this genotype is relatively uncommon among adults (7). In a case series of four patients from Kansas with particularly severe manifestations, including refractory status epilepticus and myocarditis, methylprednisolone or hydrocortisone was added to existing IVIG treatment. Nevertheless, the role of corticosteroids and IVIG in HPeV encephalitis remains uncertain and requires further investigation (6). In severe enterovirus infection in neonates, IVIG administration showed minor clinical benefits (11). Due to the severity of the clinical presentation in this case including abnormal brain MRI and abnormal aEEG, IVIG was administered.

The total duration of hospitalisation was eleven days. In a case series of severe HPeV infections among infants younger than three months, patients requiring intensive care had a mean hospital stay of 22.5 days (9).

Although favourable neurodevelopmental outcomes have been reported following HPeV encephalitis, the prognosis appears to depend on disease severity. Extensive abnormalities on brain MRI have been associated with poorer neurocognitive outcomes (4, 15). Moreover, the literature suggests that more than half of patients with severe HPeV encephalitis experience subsequent neurodevelopmental difficulties (7). In another study, neurodevelopmental impairment was identified in only 20% of infants 18 months after HPeV central nervous system infection, predominantly affecting fine and gross motor function; these abnormalities had resolved six months later. By comparison, developmental concerns were reported in 48.3% of infants following enterovirus central nervous system infection (19).

All in all, the aspect of neurodevelopment following HPeV encephalitis requires further investigation.

Although the follow-up findings in our patient have so far been reassuring, regular assessments at a specialist child development centre have been arranged to monitor his ongoing development. The optimal duration of surveillance remains uncertain. Developmental assessments at 6, 12, and 24 months after hospitalisation have been proposed (7). Prolonged follow-up may be warranted in the presence of risk factors such as PICU admission, abnormal MRI findings, infection during the neonatal period, seizures, or apnea (4). As all these risk factors were present in our patient, developmental surveillance beyond two years should be considered. Given the severity of the clinical presentation, close monitoring was arranged at our outpatient paediatric neurology clinic, with initial follow-up assessments scheduled at one, three, six and 18 months after discharge. Further assessments will be scheduled depending on the respective outcomes.

In conclusion, HPeV encephalitis should be considered in neonates and young infants presenting with fever without an identifiable source, particularly when accompanied by apnea and seizures. Despite extensive diffusion restriction, recurrent seizures, a burst-suppression background, and the need for invasive ventilation, the infant in this case showed rapid clinical recovery and no evident developmental impairment during the first six months of follow-up. However, patients with severe HPeV encephalitis are at risk of long-term neurodevelopmental impairment and should therefore receive structured, long-term developmental follow-up.

## Data Availability

The original contributions presented in the study are included in the article/Supplementary Material, further inquiries can be directed to the corresponding author.
